# Molecular Genotyping of the Human Cystic Echinococcosis in Mazandaran Province, North of Iran

**Published:** 2019

**Authors:** Zeynab HEDAYATI, Ahmad DARYANI, Shahabeddin SARVI, Shirzad GHOLAMI, Mehdi SHARIF, Majid PIRESTANI, Samira DODANGEH, Simin BARI, Sara GHOLAMI, Azadeh MIZANI, Seyed Abdollah HOSSEINI

**Affiliations:** 1. Toxoplasmosis Research Center, Mazandaran University of Medical Sciences, Sari, Iran; 2. Department of Parasitology and Medical Entomology, Faculty of Medical Sciences, Tarbiat Modares University, Tehran, Iran; 3. Student Research Committee, Mazandaran University of Medical Sciences, Sari, Iran; 4. Department of Medical Parasitology and Mycology, School of Medicine, Mazandaran University of Medical Sciences, Sari, Iran

**Keywords:** *Echinococcus granulosus*, Molecular characterization, NADH dehydrogenase 1, Iran

## Abstract

**Background:**

The larval stage of the tapeworm (cestode) *Echinococcus granulosus* is the etiological agent of hydatidosis or cystic echinococcosis, which is the zoonotic parasitic disease causing morbidity and mortality in both humans and livestock. Due to a lack of accurate data on the human isolates of *E. granulosus* in Mazandaran Province, northern Iran, the current study aimed to survey the population genetic pattern of cystic echinococcosis isolated from humans by sequencing the mitochondrial genes of NADH dehydrogenase subunit 1 (*nad1*).

**Methods:**

Overall, 47 formalin fixed paraffin-embedded tissue (FFPT) blocks were collected from patients’ files in various pathology departments of Mazandaran Province in Iran from 2003 to 2015. PCR was performed to amplify a 398bp DNA fragment of mitochondrial *nad1*. PCR products were sequenced by Bioneer Corporation (South Korea), and the resulting data were analyzed via relevant software to determine the genotypes.

**Results:**

The *nad1* gene was successfully amplified on 10 from all of the *E. granulosus* isolates. Overall, 66.6% and 33.3% of the isolates in the studied area displayed the G1 and G2–G3 genotypes, respectively.

**Conclusion:**

This study may provide the foundation for further studies in revealing the regional transmission patterns and also in designing adequate control procedures.

## Introduction

The larval stage of the tapeworm (cestode)*Echinococcus granulosus* is the etiological agent of hydatidosis or cystic echinococcosis, the zoonotic parasitic disease-causing morbidity and mortality in both humans and livestock. This disease is almost cosmopolitan with great contribution in parts of Eastern Europe, North Africa, South America, the Mediterranean regions, Central Asia, and China (1–3).

Dogs and other canines are the definitive hosts, while herbivores and omnivores act as intermediate hosts (4). Humans are accidental intermediate hosts infected by parasite eggs. The emerging larvae can spread to most organs, particularly the lung and the liver, developing and creating cysts(5).

A wide degree of variation has been described within *E. granulosus* and several well-characterized strains are recognized presently. They demonstrate remarkable variation in life cycle patterns and host specificity (6, 7). Using mitochondrial DNA (mtDNA) sequences, 10 distinct genetic strains (G1–G10) within *E. granulosus* have currently been characterized (7–15). Therefore, the species *E. granulosus*comprises five valid subspecies: *E. granulosus*sensu stricto (s.s.) (genotypes G1–G3), *E. equinus* (G4), *E. ortleppi* (G5), *E. canadensis* (genotypes G6–G8, G10), and *E. felidis* (G9) (2, 6, 16–20).

In Iran, cystic echinococcosis is recognized as one of the most important parasitic zoonoses causing significant social losses due to its high surgical incidence rate in humans as well as economic losses for the animal husbandry industry. Its occurrence is for the most part contingent upon the sheep and dog cycles.

*E. granulosus* sensu stricto (clustering genotypes G1 to G3) and *E. canadensis* (G6) have been identified in humans and domestic animals such as sheep, camels, and buffalos in different regions of Iran (21–27). Due to a lack of accurate data on the human isolates of *E. granulosus* in Mazandaran Province, the current study surveys the population genetic pattern of cystic echinococcosis isolated from humans by sequencing the mitochondrial genes of NADH dehydrogenase subunit 1 (*nad1*). The findings of the present study can have promising outcomes for the design and development in the diagnosis, treatment, vaccine production and control of the *E. granulosus* infection.

## Materials and Methods

### Collection of hydatid cysts

Forty-seven formalin fixed paraffin-embedded tissue (FFPT) blocks were collected from patient files in various pathology departments of Mazandaran Province, northern Iran from 2003 to 2015. These samples were taken from patients diagnosed with echinococcosis by either detecting protoscoleces and hooklets or PAS-positive laminated layers. Three thickness sections (6 μm) were prepared from each tissue block, placed in 1.5 ml microtubes and transferred to the parasitology laboratory at Mazandaran University, Iran.

In order to deparaffinize the sections, they were submerged in 1ml xylene at room temperature for 15 min. Following this, the samples were centrifuged at 1400 rpm for 5 min and the supernatant was removed. This procedure was carried out twice. After deparaffinization, the samples were rehydrated twice in 100% ethanol, and subsequently, once in 90%, 70% and 50% ethanol. Then, the 50% ethanol was removed (28). After the alcohol evaporated at room temperature, the DNA was extracted by adding a tissue lysing solution.

### DNA Extraction

In order to extract genomic DNA, each sample was washed with distilled water three times. Following this, the genomic DNA was extracted using the conventional manual phenol-chloroform method (24, 29).The concentration of the DNA obtained was estimated with NanoDrop-1000 and stored at −20 °C.

### Mitochondrial PCR Amplification

PCR was performed to amplify a 398bp DNA fragment of the mitochondrial NADH dehydrogenase 1 (*nad1*) as reported previously (23). The primer sequences utilized were 5′ CGTAGGTATGTTGGTTTGTTTGGT3′ (Forward) and 5′ CCATAATCAAATGGCGTACGAT3′ (Reverse).

The PCR reaction carried out in total volume of20μlconsisted of2 μl PCR buffer (10×), 2 μl dNTPs, 1 μl MgCl2, 1 μl of each primer, 1 unit Taq DNA polymerase, 11μldistilled water, and 1 μl DNA. The PCR protocol was as follows: The thermal cycler for *nad1* primer was set for 94 °C (3 min) for initial denaturation and denaturation at 94 °C (30 sec), annealing at 53 °C(30 sec), extension at 72 °C (30sec) in 35 cycles, and the final extension 72°C(5 min). The PCR products were separated with electrophoresis on a 1.7% agarose gel mixed with safe stain and observed on a UV transilluminator.

### Sequencing and phylogenetic analysis

PCR products were sequenced by Bioneer Corporation (South Korea). Nucleotide sequence analysis was performed with BLAST (http://www.ncbi.nlm.nih.gov), whereas alignments were undertaken using software packages such as ClustalX and BioEdit. The *nad1* sequences of the representative isolates were submitted to the GenBank. A phylogenetic tree was obtained by using the Molecular Evolutionary Genetics Analysis (Mega7) software package. The dendogram was drawn by comparing the sequences obtained in the present study and reference sequences of all described *E. granulosus*genotypes (G1–G10) in the GenBank, and *Taenia saginata* was considered as the outgroup in the model.

The evolutionary history was obtained by means of the maximum likelihood (ML) approach based on the Kimura 2-parameter model. Primary tree(s) were obtained automatically by applying the neighbor-joining (NJ) method to a matrix of pairwise distances estimated utilizing the maximum composite likelihood method, and then choosing the topology with a higher log likelihood value. The representative tree was drawn to scale.

### Ethics statement

Ethical approval of this study was obtained from the Ethics Committee in Mazandaran University of Medical Sciences, Mazandaran, Iran.

## Results

**Fig. 1:**
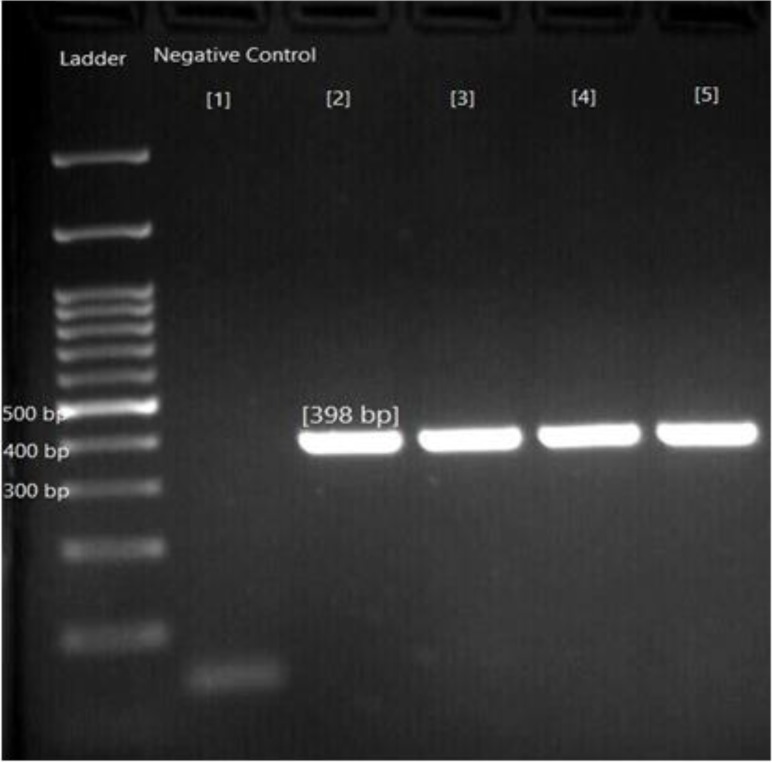
PCR amplified *nad1* fragments from human isolates of *E. granulosus* from Mazandaran, Iran. Ladder 100bp, lan1: negative control, lane 2–5: Positive sample

The *nad1* mitochondrial gene was successfully amplified 10 of 47 FFPT samples (21.27%) of histologically confirmed *E. granulosus*gave positive results with the PCR,37 (78.72%) showed no reaction. For all amplicons, the consensus length of 398bp nucleotides was obtained (Fig. 1).

The DNA sequencing was done on these amplicons for the *nad1* gene. Overall, 66.6% and 33.3% of the isolates displayed the G1 and G2–G3 genotypes, respectively. In this study, 6 haplotypes (E7–E12) were determined based on the sequence region. The sequences from the *E. granulosus nad1* (398 bp) isolates were submitted to GenBank under accession numbers MG693717–MG693722.

**Fig. 2:**
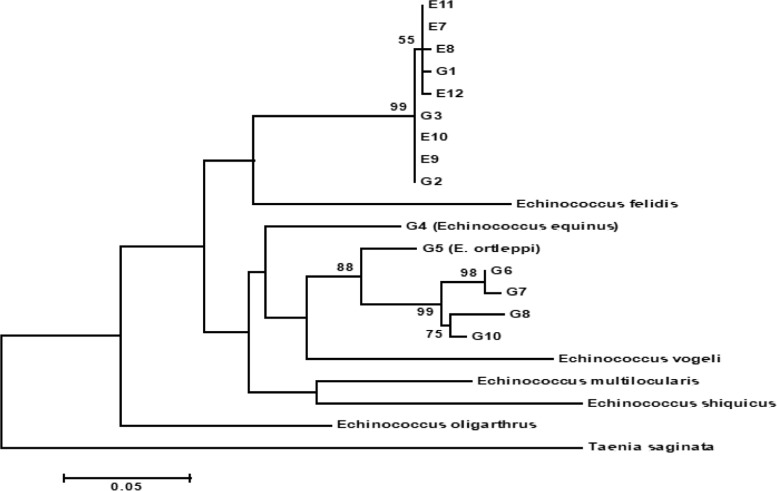
Genetic relationships of *E. granulosus* human isolates from Mazandaran Province (North of Iran) and reference sequences for *E. granulosus* sensu stricto and *Taenia saginata* as the out group. The relationships were inferred based on the phylogenetic analysis of the concatenated *nad1* sequence data (E7–E12). All haplotypes represent genotypes G1–G3 (G1–G3 complex, *E. granulosus* sensu stricto)

Phylogenetic analysis of the sequences ML is demonstrated in Fig. 2. All of the sequences were grouped into a distinct cluster related to the G1–G3 complex with relevant reference sequences.

## Discussion

Echinococcosis is a clinically and epidemiologically important health problem in many countries, particularly those with large populations of livestock (30). Based on reports, this disease is endemic in the majority of provinces in Iran (31–34).

In this investigation, complete *nad1* mitochondrial gene sequences were employed to analyze the genetic characterization of *E. granulosus* in Mazandaran Province. For a better understanding of the processes of intra and interspecific gene flow, research on the population genetic characterization of *E. granulosus* is essential. It can also provide groundwork for future epidemiological studies on the transmission dynamics of this parasite in animal hosts (35–39).

Based on random-effects meta-analysis, the prevalence of human cystic echinococcosis (HCE) has been reported 5.0% in Iran (40). Moreover, an approximate 1% of all surgeries in Iran is caused by hydatidosis (41).

Several studies on mitochondrial and ribosomal genomes have revealed the presence of two distinct strains, namely sheep (G1) and camel (G6) in Iran (22, 42–44). Sharbatkhori et al. (22, 24) identified the buffalo strain (G3) from Iranian camels using mitochondrial sequencing of partial *nad1* and cox1 for the first time (22, 23). Later, genotype G3 was also detected from buffalos (27), cattle, sheep, and camels (25, 44) in Iran.

In spite of suitable weather conditions for *E. granulosus* infection in Mazandaran Province, no study has yet been conducted for its characterization. Therefore, this paper presents the first molecular identification of human cystic echinococcosis using mitochondrial loci in Mazandaran Province.

The present study reveals the G1–G3 complex (*E. granulosus* sensu stricto) by sequencing mitochondrial *nad1* in human isolates from Mazandaran Province. A previous study conducted in Golestan Province, adjacent to Mazandaran, using DNA regions cox1 and *nad1* showed that all classified cyst isolates from humans and ruminants belonged to the G1–G3 complex(*E. granulosus*sensustricto) (23).Another study, usingITS1-RFLP, indicated the presence of G1 genotype in isolates originating from human, cattle and sheep isolates in Tabriz, north westernIran (45). In this study, G1 was reported as the most prevalent genotype among the isolates. The sheep–dog cycle can be suggested as the dominant cycle in CE. The G1 genotype of *E. granulosus* is the most frequent genotype detected in both animal and human isolates throughout the world (46–50). However, in some countries in North Africa, Sudan and Mauritania, G6 has been demonstrated as the most common genotype in both animal (cattle, camels) and human isolates (51, 52).

## Conclusion

Based on the concatenated sequence of *nad1*, there were 6 different haplotypes (E7–E12) in the present study. The findings of this study are consistent with previous studies from Iran and demonstrate *E. granulosus* strain G1 as the predominant haplotype.G2–G3 genotypes of *E. granulosus* were also identified among the hydatidosis isolates (33.3%).

This study may provide the foundation for further studies in revealing the regional transmission patterns and also in designing adequate control procedures. To further assess the diffusion of *E. granulosus* from Mazandaran Province to the surrounding regions, more studies are required.
